# Making Human Neurons from Stem Cells after Spinal Cord Injury

**DOI:** 10.1371/journal.pmed.0040048

**Published:** 2007-02-13

**Authors:** Natalia Abramova Lowry, Sally Temple

## Abstract

A new study by Yan and colleagues makes an important contribution to research on human spinal cord stem cells.

Spinal cord injury (SCI) has been recognized as one of the conditions for which stem cell transplantation might first prove beneficial [1]. After SCI, loss of localized myelinating oligodendrocytes and grey matter neurons occurs, with glial scar formation and degeneration of both descending and ascending axons (Figure 1). Replacement of oligodendrocytes to promote remyelination or of neurons to assuage neuronal loss and damage through establishment of relay circuitry or release of trophic factors, are possibilities for stem cell transplantation intervention. Scientists and clinicians recognize the need to move cautiously toward cell replacement goals, as damaging results due to premature clinical testing would be devastating for patients and the emerging stem cell neural repair field. Animal studies need to address fundamental questions—Which is the best cell source for neuron or oligodendrocyte replacement, what is the best location for transplantation, and what is the best time-course after injury?

**Figure 1:**
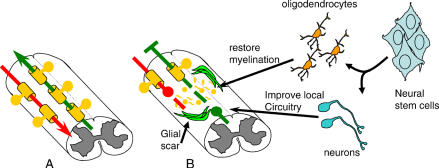
Normal and Injured Spinal Cord (A) Normal spinal cord with examples of ascending (green) and descending (red) pathways. (B) After SCI, degeneration of both descending and ascending axons occurs with glial scar formation and loss of local myelinating oligodendrocytes and grey matter neurons. Stem cells could provide growth factors beneficial to SCI repair, or help replace lost myelin or circuitry.

## Embryonic Spinal Cord Stem Cells Are Closest to the Goalpost

Embryonic stem cells can be pushed to generate cells with characteristics of spinal cord neurons and oligodendrocytes, and studies are ongoing to establish the breadth of spinal cord cell types that can be produced, their long-term stability, and their functional authenticity. Another stem cell source that is being pursued, and that may in fact be closer to the goal of producing a variety of bone fide spinal cord cells, is stem cells from the spinal cord itself. These are a subclass of neural stem cells (NSCs) and as such are restricted to generating neural tissues, which is a notable advantage over embryonic stem cells, and have the further advantage of being regionally specified to produce spinal cord progeny. Stem cells can be isolated from spinal cord from embryonic through adult stages [2–4]; however, early embryonic stages most readily generate a wide array of spinal cord neurons and glia: NSCs restrict their developmental potency over time, and unfortunately we do not currently know how to reverse the NSC aging process.

## Stem Cells Used for SCI

A recent terrific review [5] summarizes the state of stem cell transplantation for SCI. Previous studies on implanting embryonic NSCs after SCI show that these cells can make oligodendrocytes effectively in vivo; however, neuron production is notably poor. These results have led to the idea that the adult SCI environment does not allow NSCs to differentiate efficiently into neurons. Starting with a more differentiated cell population such as NRPs—restricted progenitors for neurons—allows neuron production [6–8], perhaps because these cells no longer need environmental instruction to attain the neuronal fate. While, GRPs—restricted progenitors for glia—and NRPs could be valuable for producing specific types of progeny, it is still appealing to consider use of NSCs that could, in theory, generate a wider variety of neurons and glia, potentially tailoring output according to the specific cell needs of an individual SCI situation. Hence, it is somewhat disappointing that prior NSC studies have not led to more neuron production in vivo. However, it is important to realize that of the prior NSC SCI studies (approximately 30), most actually used brain NSCs. Only three used NSCs from embryonic spinal cord, two from rat [9,10], and one from human [3]. When we consider the dearth of spinal cord NSC studies, especially in humans, it is perhaps premature to view the adult injured cord as lacking crucial differentiation instructions.

**Glossary**

**ChAT:** Choline acetyl transferase, present in motor neurons

**GABAergic:** A marker indicative of inhibitory neurons

**GFAP:** A marker of astrocytes also expressed by some NSCs

**GRP:** Restricted progenitor for glia

**Nestin:** A marker of progenitor cells

**NeuN:** A nuclear marker of more mature neurons expressed by many neuron types

**NRP:** Restricted progenitor for neurons

**NSC:** Multipotent, self-renewing cell producing neurons and glia, but not non-neural cells

**TUJ1:** An early marker of neurons

## Filling the Gap with Human Spinal Cord Stem Cells

**Figure 2:**
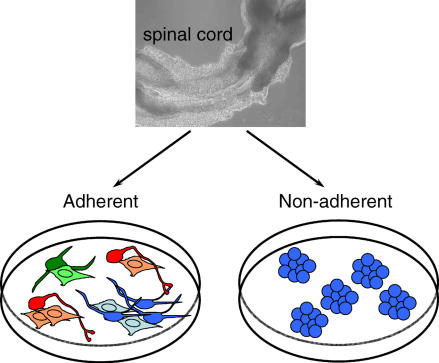
Spinal Cord Progenitors Isolated from the Cord and Grown in Tissue Culture as Adherent Cultures or Nonadherent Nanospheres More heterogeneity is seen after adherent culture compared to neurosphere culture.

The need for human spinal cord stem cell studies alone makes the paper by Yan et al. [11] an important contribution. Moreover, it is the first to assess the potential of human embryonic spinal cord cells expanded in adherent rather than neurosphere culture. Most of the prior NSC studies used cells expanded using neurosphere generation, in which nonadherent cells proliferate to form multicell spheres. This is a standard method for propagating stem cells [12,13], but only very few cells can proliferate under these conditions, and this technique might select for a particular subpopulation (and potentially a gliogenic subpopulation) of stem cells [14]. In contrast, adherent culture allows growth of a larger and more heterogeneous population of starting cells (Figure 2) [15,16], as well as providing different surface signaling during the expansion phase that could well impact subsequent cell behavior. Here Yan et al. investigate the fate of adherent cultured human embryonic spinal cord stem cells in an adult SCI environment.

Spinal cord cells were obtained from cervical and upper thoracic spinal cord of an eight-week-old human embryo and expanded in monolayer culture in defined medium with the mitogen FGF2 (a member of the fibroblast growth factor family). They could be propagated for at least 20 passages and frozen and thawed with good recovery. Most of the cells expressed the progenitor marker Nestin, but upon withdrawal of FGF2, the cells differentiated, and approximately 50% became neurons (expressing the neuronal marker MAP2). Cells in differentiated cultures expressed neurotrophic factors and neuregulins, indicating that they could produce potentially useful agents for SCI.

Two types of injury were examined: extraspinal avulsion of L4 and L5 spinal nerves, and excititoxic lesion at the L4–L5 levels. Passage 10–12 cells were used in the study and FGF2 was removed from the culture up to 24 h before the cells were transplanted. Two weeks postlesion, animals received four injections of 10^5^ spinal cord stem cells, each in 0.5 ml suspension, into ventral L4 and L5 on the left side 1 mm lateral to the midline.

## Abundant Human Neuron Production in the Adult Spinal Cord

The implanted cells showed robust engraftment and good long-term survival. NSCs migrated away from the initial grafting sites and populated both white and gray matter, as well as portions of dorsal roots in avulsion cases. 3%–5% of cells remained mitotically active (i.e., were positive for the proliferating cell marker Ki67). Most surprising was the finding that over 70% of cells in dorsal or ventral horn of L4–L5 expressed the neuronal marker TUJ1. 11%–14% of the cells were Nestin positive, and only 5% expressed the astrocyte marker GFAP. Of cells residing in white matter, approximately 60% were neurons and 16%–20% were positive for the astroglial marker GFAP. Very few cells became oligodendrocytes (9% in grey matter and 12% in white matter). The authors further defined the neuronal phenotypes with extensive immunoanalysis. Many expressed the more mature marker NeuN, and most developed GABAergic phenotypes (see Box 1 for glossary). Moreover, these cells were contacted by GABAergic terminals from graft and host neurons and glutamatergic terminals from the host. Less than 1% of cells were ChAT positive, indicating little motor neuron generation. There is evidence for process outgrowth from the engrafted neurons, and for synaptic connections with host motor neurons.

To our knowledge this study is the first to show that adult human spinal cord stem cells can be expanded in adherent culture without genetic modification to generate large numbers of cells sufficient for transplantation, and the first NSC study to find significant neuron generation in the injured adult spinal cord. The authors conclude that the adult spinal cord environment can in fact encourage spinal cord stem cells to make new neurons efficiently. This study also indicates that differences in the culture technique, such as the cell expansion method, can have a major impact on NSC behavior after transplantation.

## Future Studies

The production of so many neurons in the adult in vivo reported here has been an elusive goal of stem cell studies for years. Given the significance of this finding, it will be critical to verify that these cells are bone fide functional neurons using electrophysiological recording, beyond the use of immunomarkers described in the paper. It will also be crucial to repeat this work using different primary lines established from human embryonic spinal cord. If this result is verified, then future studies should investigate whether diverse subtypes of spinal cord neurons, such as motor neurons, can be produced—for example, if an earlier embryonic spinal cord source is used, or if the cells are treated with growth factors such as Shh prior to transplantation—generating large quantities of motor neurons will be beneficial for treating neurodegenerative diseases such as ALS (amyotrophic lateral sclerosis). It will also be important to determine whether significant neuron production is obtained after other experimental types of SCI—e.g., the contusion model [17]—which create the type of injuries closer physiologically to those typically seen in human patients [18]. Additionally, it should be noted that to study the behavior of human cells in the rat experimental SCI models, the authors had to immunosuppress the animals or use nude hosts. Since SCI in rodents is usually accompanied by substantial infl ammation, the use of immunosuppressed animals is not a perfect model for study of transplanted cell behavior. This, however, is a general problem encountered when studying the behavior of human cells in animal models. It seems unlikely that effective neuron production could have resulted from immunosuppression, but that is certainly something that could be examined. Finally, since behavioral improvement is the ultimate measure of the success of the transplantation treatment, further studies should include behavioral analysis of the experimental subjects.

## Abbreviations

NSC: neural stem cell
SCI: spinal cord injury
